# Supplementary material for: Perceived Barriers and Facilitators in Cardiovascular Risk Management in Colombia: A Qualitative Analysis of the RE-HOPE Study
Source: Int J Environ Res Public Health. 2025 Jul 31;22(8):1199. doi: 10.3390/ijerph22081199 (PMC12386406; doi:10.3390/ijerph22081199)
Supplement: Supplementary file 1 [file ijerph-22-01199-s001.zip › ijerph-3716444-supplementary.pdf]

Supplementary Material

Supplemental Table 1. Operational definitions of codes for interpretation.

Code	Operational definitions
Strategies	Set of measures adopted or created for the prevention and management of cardiovascular risk.
Resources	Instruments, means or tools used for cardiovascular risk management. Human, economic, infrastructure and technological resources, among others, were included.
Access	Conditions or circumstances that favor or do not favor admission for the provision of health services in primary and complementary care.
Risk measurement	Sources of information to know the magnitude of cardiovascular events or risk stakeholders.
Cross-sector collaboration	Concurrence of actions from other sectors, including the health sector, for the prevention and management of cardiovascular risk.
Articulation	It describes the interaction between different stakeholders in the health sector such as patients, community agents, territorial entities, providers and insurers.
Stewardship	It indicates the leadership capacity of the state and its institutions to guide, agree on and execute actions that promote the well-being of the population

Supplemental Table 2. Participants' quotes for each category.

**Code category**	**Quotes**
Strategies	• *"Community groups previously employed trained health educators and physical activity facilitators. Our work began locally before scaling department-wide through the Santander en Movimiento program, which operated successfully for more than 10 years" Woman, 57, territorial entities.*
Stakeholders	• *"The patient risk identification process was initially implemented with a 2023 completion target, intended to generate consolidated data for insurers to develop evidence-based population intervention plans." Woman, 39, Healthcare Insurers.*
	• *"The EPS door-to-door initiative demonstrates particular value in our experience. While our facility faces challenges in patient retrieval, the most we can do is call, and many times it is lost, the insurer's home visits prove more effective, especially for elderly patients who respond better to printed appointment reminders and personal engagement." Woman, 50, healthcare providers.*
Community-based actions	• *"Health promotion efforts should prioritize lifestyle modifications, particularly through physical activity promotion and evidence-based nutritional guidance." Woman, 36, healthcare providers.*

Resources	• *"All activities within the collective intervention plan have been progressively implemented, particularly focusing on resource-limited municipalities. This initiative specifically supports early diagnosis of cardiovascular risk populations through complementary services." Woman, 41, Territorial Entities.*
	• *"Dedicated personnel must be assigned to these activities. As healthcare providers, we already carry substantial clinical responsibilities with high time demands, making additional tasks unsustainable without proper staffing allocations." Woman, 41, Nurse, healthcare providers.*
Service fragmentation	• *"Our model's key advantage lies in its structured coordination mechanism: a dedicated cardiovascular risk professional within the healthcare insurers facilitates continuous collaboration between primary and complementary providers. This tripartite integration has improved care pathway efficiency, with measurable outcomes already emerging. Notably, our first-level training program from the complementary care component represents one of the most successful current initiatives for this patient cohort." Woman, 39, Healthcare Insurers.*
	• *"The IMAP (information system) team includes dedicated analysts and a supervising professional who track medication adherence and other key indicators. These monitoring reports inform our decisions as the contracting authority for providers." Woman, 36, Healthcare Insurers*
Access	• *"Our cardiovascular risk population is predominantly elderly, with limited education and minimal social support networks. These compounding factors, particularly low health literacy, are so significant that many patients don't even understand their prescribed medications." Woman, 52, Physician, healthcare providers.*
	• *"Frequent medication stockouts create dangerous care gaps: patients must return to physicians for alternative prescriptions, wait indefinitely for deliveries from the pharmacy, or ultimately disengage from care entirely. This systemic fragility particularly disadvantages vulnerable populations." Woman, 26 years old, Nursing Assistant, healthcare providers.*
Cross-sector collaboration	• *"Significant systemic challenges exist in coordinating with non-health sectors like infrastructure, sports, and education. A prime example is the CERS (Healthy Cities, Environments and Ruralities) strategy, its implementation requires collaboration on sports infrastructure that falls outside the health sector's jurisdiction, creating substantial interdepartmental coordination barriers." Woman, 57, territorial entities.*
	• *"While we recognize the need for substantial improvements in cardiovascular risk reduction, success ultimately depends on factors beyond our direct control, social determinants, and systemic conditions that collectively influence population health outcomes." Woman, 31, Nurse, healthcare providers.*
Risk measurement	• *"Program success is ultimately demonstrated through outcome indicators, these metrics provide the most objective evidence of intervention effectiveness." Woman, 39, territorial entities.*
Stewardship	• *"There are persistent systemic barriers at the departmental government level that hinder effective implementation." Woman, 57, territorial entities.*

	• *"We require dedicated personnel and policy clarity, not just more strategies. Territorial entities must prioritize and operationalize specific interventions through committed, focused action." Woman, 57, territorial entities.*

Supplemental Figure 1. Network diagram illustrating the interrelationships among institutional stakeholders as they evolve through emerging subcategories.

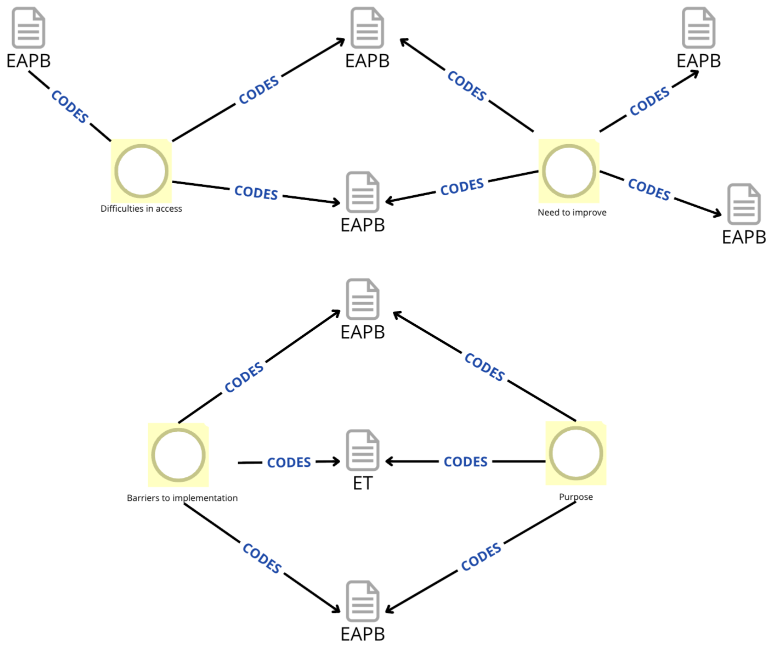

EAPB: Health Benefit Plan Management Companies. IPS – Health-service Provider Institutions. ET: Territorial Entities
